# Instability and Stasis Among the Microbiome of Seagrass Leaves, Roots and Rhizomes, and Nearby Sediments Within a Natural pH Gradient

**DOI:** 10.1007/s00248-021-01867-9

**Published:** 2021-10-01

**Authors:** Raymond B. Banister, Melbert T. Schwarz, Maoz Fine, Kim B. Ritchie, Erinn M. Muller

**Affiliations:** 1 Mote Marine Laboratory, Coral Health and Disease Program, Sarasota, FL USA; 2 Institute for Global Ecology, Florida Institute of Technology, 150, W University Blvd, Melbourne, FL 32901 USA; 3 The Goodman Faculty of Life Sciences, Bar-Ilan University, 52900 Ramat Gan, Israel; 4 The Interuniversity Institute for Marine Science, P.O.B. 469, 88103 Eilat, Israel; 5 Department of Natural Sciences, University of South Carolina Beaufort, 801, Carteret St., Beaufort, SC 29906 USA

**Keywords:** Seagrass, Microbiome, Vulcano, Natural pH gradient, *Cymodocea nodosa*, Bacterial community

## Abstract

Seagrass meadows are hotspots of biodiversity with considerable economic and ecological value. The health of seagrass ecosystems is influenced in part by the makeup and stability of their microbiome, but microbiome composition can be sensitive to environmental change such as nutrient availability, elevated temperatures, and reduced pH. The objective of the present study was to characterize the bacterial community of the leaves, bulk samples of roots and rhizomes, and proximal sediment of the seagrass species *Cymodocea nodosa* along the natural pH gradient of Levante Bay, Vulcano Island, Italy. The bacterial community was determined by characterizing the 16S rRNA amplicon sequencing and analyzing the operational taxonomic unit classification of bacterial DNA within samples. Statistical analyses were used to explore how life-long exposure to different pH/*p*CO_2_ conditions may be associated with significant differences in microbial communities, dominant bacterial classes, and microbial diversity within each plant section and sediment. The microbiome of *C. nodosa* significantly differed among all sample types and site-specific differences were detected within sediment and root/rhizome microbial communities, but not the leaves. These results show that *C. nodosa* leaves have a consistent microbial community even across a pH range of 8.15 to 6.05. The ability for *C. nodosa* to regulate and maintain microbial structure may indicate a semblance of resilience within these vital ecosystems under projected changes in environmental conditions such as ocean acidification.

**Supplementary Information:**

The online version contains supplementary material available at 10.1007/s00248-021-01867-9.

## Introduction

Seagrass meadows cover 300,000 to 600,000 km^2^ of coastline worldwide [1, 2] and are considered a hotspot of biodiversity. Along with supporting vast biodiversity, seagrasses are considered economically and ecologically important. They play a critical role in recycling nutrients, providing food and shelter to a multitude of marine organisms, and stabilizing sediments [3]. The presence of healthy seagrass meadows reduces exposure of bacterial pathogens to humans, fishes, and invertebrates [4]. A vital component of seagrass meadows is the presence and health of their microbiome. How seagrass microbiomes will change under future ocean conditions and the further ecological effects of these potential changes are unknown [5]. Seagrass coverage has faced an accelerated decline under current ocean conditions [6] emphasizing the need to understand seagrass health under changing environmental scenarios before further loss occurs.

A functioning microbiome is vital to the physiology, ecology, and biogeochemistry of seagrass habitats [7], but the plant–microbe relationship is complex and not well understood under changing ocean conditions. Bacteria in the form of an epiphytic biofilm on seagrass blades are essential components that can influence the settlement of accumulating microbes and other epiphytic organisms. Conversely, these biofilms can affect light availability through physical shading and potentially pose a threat if not regulated by the competing microbial community [8, 9]. Growing research on the plant–microbe mutualistic relationship suggests that some bacteria within the seagrass leaf microbiome aid in plant growth through nitrogen fixation [10–14], limit growth of competitive algal epiphytes, and metabolize potentially harmful metabolic waste produced by the plant such as methanol and ethanol [15, 16]. Beneficial bacterial associations are not unique to the seagrass leaves, however, and can be found in the rhizosphere of seagrasses, providing similar functions [3, 15, 17–24].

The rhizosphere of seagrasses has a unique bacterial community that is typically important within the sulfur cycle and aids in shaping the overall health of the seagrass meadow [3, 15]. The rhizosphere is important for nutrient uptake and shifts in the microbiome could impact the conversion of organic material into essential nutrients and compounds [17–21]. Additionally, the roots and rhizomes self-regulate via sulfur detoxification under stressful environmental conditions [22–24]. The driving factors that cause variation among rhizosphere microbial communities under stable condition changes with the scale that rhizospheres are compared. At a local scale, microbial variation within the rhizosphere is due to the differences in light availability that supply organic nutrients from the leaves to the rhizosphere through photosynthesis. Conversely, at a regional scale, the variation in nutrient levels and soil toxicity becomes a greater driver of variation in the rhizosphere’s microbial community [3].

Sediments within seagrass meadows are often an extreme environment with anoxic conditions commonly produced through the mineralization of organic material throughout the benthic layer [25–27]. In addition, waterlogged sediment prevents oxygen diffusion to the water column further promoting anoxic conditions. This, in addition to sulfate reduction from microbes present in the sediment and rhizosphere, also results in a highly acidic environment within marine sediments [28, 29]. The unique bacterial community within the extreme environments of the sediment is partly responsible for nitrification and denitrification as well [30, 31]. Nitrification–denitrification processes are essential in seagrass ecosystems to prevent the buildup of organic matter byproducts in the sediment. For example, particular bacteria compete for ammonia and break down NH_4_^+^ into NO_2_^−^ [32, 33], mitigating eutrophication prompted by nitrogen buildup [6, 34]. Seagrass can alter its environment and store valuable nutrients within the sediment [35–37] and as environmental conditions continue to change, these vital processes could be impacted, changing the functional dynamics and overall health of seagrass ecosystems [31].

Global oceans are changing. By 2100, the average atmospheric CO_2_ is anticipated to reach 985 ± 97 ppm (full-range 794–1142 ppm), which will concomitantly result in higher oceanic *p*CO_2_ conditions [38]. High *p*CO_2,_ along with a rise in bicarbonate, allows for optimal photosynthesis [39]. Although this may be beneficial in the context of photosynthetic enhancement, seagrasses are complex organisms and a rise in *p*CO_2_ may not be advantageous in all aspects. A study characterizing phenol production in marine plants showed a decline in concentrations of phenolic protective substance with increasing *p*CO_2_ levels [40]. Phenol production serves as an herbivory deterrent, digestion reducer, and antifoulant [41–50]. Thus, reduction in phenol production could result in higher grazing rates within areas with high *p*CO_2_, such as near CO_2_ vents, and an overall negative impact on seagrasses. At extreme levels of *p*CO_2_, however, Borell et al. (2013) showed that increased *p*CO_2_ levels changed the palatability of macroalgae reducing the grazing rates of herbivory. The change in palatability was due to an increase of secondary metabolites (dimethylsulfoniopropionate) [51]. High levels of *p*CO_2_ are also associated with a disruption in the microbiome of marine organisms. For example, seagrass within naturally high *p*CO_2_ conditions near Papua New Guinea harbored more potentially pathogenic bacteria compared with those within lower *p*CO_2_ sites [39]. On the other hand, *Caulerpa*, an invasive macroalgae, showed a stable diversity and species richness within its microbiome under high *p*CO_2_ conditions suggesting that this species may be resistent or even potentially benefit from changing ocean conditions [52]. The presence of carbon concentrating mechanisms (CCM) within *Caulerpa*’s chloroplasts aids in intracellular *p*CO_2_ regulation under high *p*CO_2_ conditions, giving them a competitive advantage over macroalgae that lack a CCM [52]. The known effects of high *p*CO2 and, concomitantly, low pH on seagrasses and their microbial communities still require further investigation as elevated *p*CO_2_ levels potentially pose a threat to seagrass ecosystems.

The objective of the present study was to characterize the bacterial communities associated with seagrass leaves, bulk samples of roots and rhizomes, and the proximal sediments found along the natural pH gradient of Levante Bay, Vulcano Island, Italy. There are few available study sites that generally mimic current and anticipated extreme conditions within one naturally occurring community. Volcanic vents contain minimal confounding variables and a natural pH gradient that allows for characterizing the present and potentially future bacterial communities of marine organisms such as seagrasses. Levante Bay located at Vulcano Island, Italy, provided a natural pH gradient to study microbial differences in a seagrass community from long-term exposure to different environmental conditions.

## Methods

### Site Characteristics and Sample Collection

**Fig. 1:**
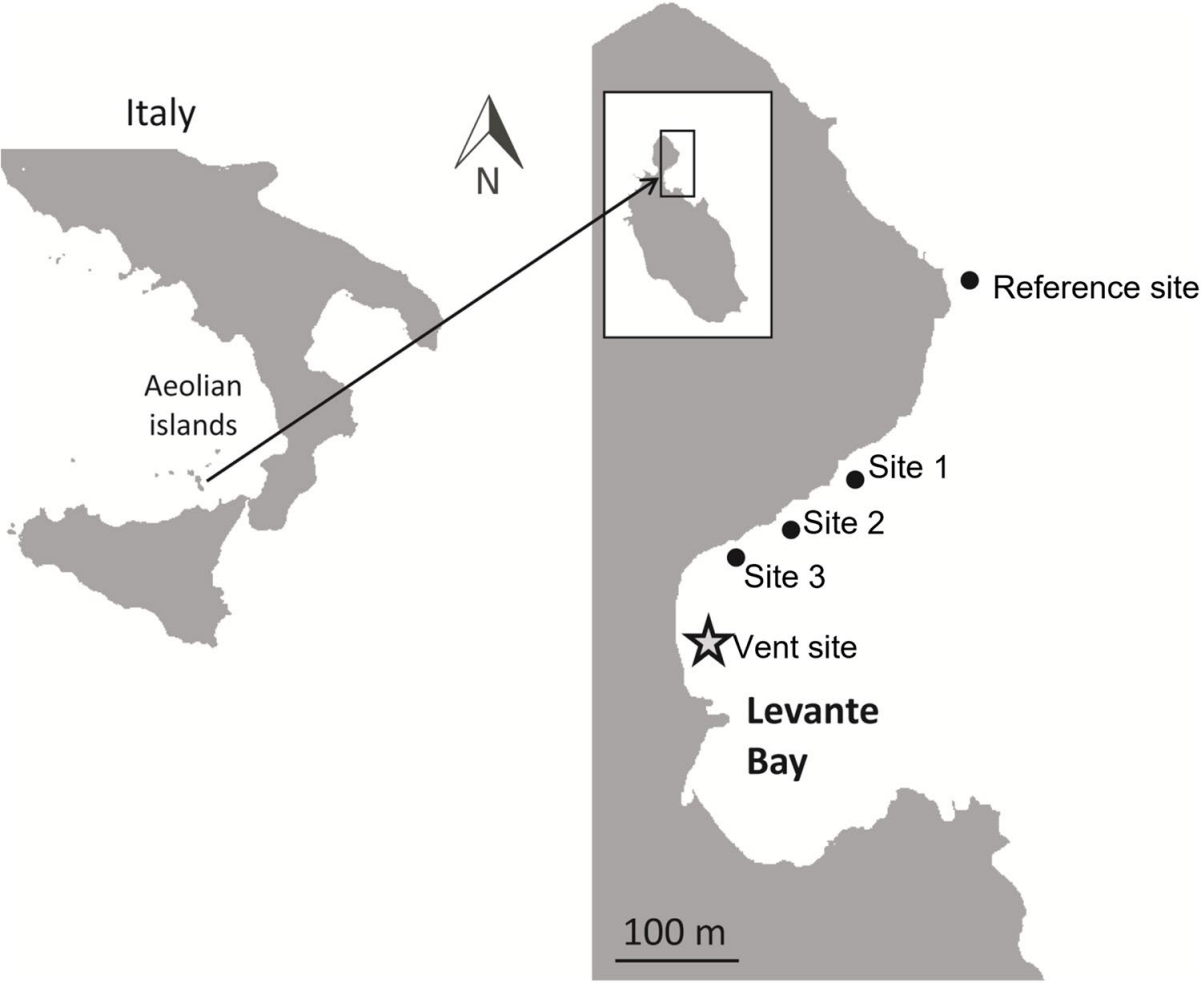
Map of sites sampled along the shoreline of Levante Bay, Vulcano Italy. Figure modified from Horwitz et al. (2015)

Leaves and bulk samples of roots and rhizomes from the seagrass *Cymodocea nodosa* and associated sediment samples were collected on June 21, 2013, in Levante Bay, Vulcano Island (38° 25′ N, 14° 57′ E), NE of Sicily, Italy. Levante Bay contains a natural seawater pH gradient ranging from 6.05 to 8.15 due to the presence of a shallow-water submarine volcanic CO_2_ vent [53]. For the present study, a total of five sites were established within Levante Bay: the reference site (500 m northeast of the vent; pH 8.15), site 1 (390 m from the vent, pH 8.03), site 2 (300 m from the vent, pH 7.86), site 3 (240 m from the vent, pH 7.44), and the vent site (15 m from the vent; pH 6.05; Fig. 1). pH_NBS_ was measured at the time of sampling using a YSI 556 handheld multimeter (YSI Inc., Yellow Springs, OH, USA). Our study did not measure other water quality information during the sampling period, but several other studies have characterized this study site. To date, there is no literature published on the trace elements along the gradient at Vulcano Island. However, two studies have published records on total alkalinity, *p*CO_2_, filamentous algae, suspended particulate organic matter, macroalgae, microbial mats, dry bulk density of sediment (g cm^−3^), porosity (φ), silt and clay (%), δ13C (‰), and C/N (Supplemental Table 1) [54, 55]. Although these two studies did not sample the same locations as the present study, the sites representing extreme conditions were located approximate to our study’s site 3 and their control site was approximate to our site 1 [54, 55].

Three individual clumps of *C. nodosa*, which contained (1) leaves, and (2) roots and rhizomes, were collected at each site (one ramet per clump). Root and rhizomes have known differences in microbial composition [56, 57]. However, for the purposes of this study, root and rhizomes were collected in bulk for analysis. In addition to the seagrass samples, three scoops, each approximately 1 g in size, of surface sediment (within ~ 1–2 cm of the surface) were taken at each site within 0.5 m of the seagrass sample. Each sample was haphazardly selected at approximately 2 m depth and approximately 2 m apart to avoid sampling clones. The samples from each of five sites (leaves, roots, and sediment *n* = 3 per site) were stored separately in the RNA stabilization reagent RNA*later* (Qiagen®) and then flash-frozen using liquid nitrogen. Enough RNA later was used to completely submerge all sample types. Although sediments were stored in separate sterile centrifuge tubes, the leaves and roots and rhizomes of the seagrasses of each ramet were not separated until sample processing occurred. Samples were transported back to Mote Marine Laboratory (Sarasota, FL, USA) via a liquid nitrogen dry shipper and stored at − 80 °C until further analysis.

### DNA Extraction and Sequencing

Nucleic acid extraction for all samples was carried out using the commercially available PowerSoil® DNA isolation kit (Qiagen®). Sediment samples were processed as per the manufacturer’s instructions. A modified protocol was used for seagrass tissues. Specific tissues (i.e., leaves and the combination of roots and rhizomes) were separated under a sterile hood using sterile scalpels. The initial lysing step for the isolation kit required both a lysing solution and mechanical bead beating for 10 min, which was extended to 1 h in order to maximize DNA yields. The initial bead beating time of 10 min, resulted in an average DNA yield of 5.4 ng/µL and a range of 0–29 ng/µL with 5 samples < 1 ng/µL. The elongated bead beating increased the average DNA yield to 8.2 ng/µL and improved the range to 2.4–29 ng/µL. Samples were not periodically cooled on ice during the elongated vortexing period; however, the average DNA strand length of our sequences (~ 450 bp) showed formidable quality even after the extended bead beating time. Extracted DNA was stored at − 20 °C until 16S rRNA amplicon sequencing. Bacterial communities of each sample were analyzed using 454 pyrosequencing of the 16S rRNA gene. Samples were sequenced by MRDNA (www.mrdnalab.com, MRDNA, Shallowater, TX, USA) with a single-step 30 cycle PCR, using primers specific for the 16S gene (27F and 519R). Samples were then sequenced using a Roche 454 FLX titanium sequencer as per the manufacturer’s guidelines.

### Sequence Analysis

Amplicon reads were analyzed by the MRDNA lab (www.mrdnalab.com, MR DNA, Shallowater, TX, USA). MR DNA trimming was completed after an FLX sequencing run followed by a quality filtering of any reads less than 150 bp. From the amplicon reads, quality sequences were binned into OTUs (operational taxonomic units) based on sequence similarity with a 97% similarity cutoff. OTUs were classified at all taxonomic levels using the curated NCBI database (www.ncbi.nlm.nih.gov) [52]. Classification of OTUs was determined using percent homology and identified to the lowest possible taxonomic level based on Supplemental Table 2. The 16S rRNA sequences analyzed in this study were deposited to NCBI PRJNA623005.

### Statistical Analyses

Only sequence reads identified as bacteria were processed through the statistical analyses. Bacterial community data were rarefied to 3036 reads per sample prior to analysis. Rarefication was chosen based on the lowest number of reads per sample within the data set (3036 reads). Community analyses were then conducted at the operational taxonomic unit (OTU) level. A permutational multivariate analysis of variance (PERMANOVA) with 9999 permutations [58] was used to determine whether there were differences in microbial communities among sample types (i.e., leaves, roots and rhizomes, and sediment samples). PERMANOVA was conducted using the vegan package in R [59]. Community profiles among sites were then visualized using non-metric multidimensional scaling (NMDS) using the Bray–Curtis dissimilarity index [60] with the metaMDS function from the vegan package in R [59]. PERMANOVA was also used to compare the bacterial community counts among sites within each sample type. When significant PERMANOVAs were detected, a pairwise PERMANOVA with a Bonferroni correction was conducted.

A Kruskal–Wallis (KW) test was used to determine whether there were differences within dominant bacterial classes (i.e., > 3% relative abundance within at least one sample) among sites within each sample type. If a significant difference was determined using the KW test, the analysis was proceeded with a Bonferroni-corrected Dunn’s test for post hoc comparison within the dunn. test package in R [61] to determine significant differences among sites. When significant differences were determined within the dominant classes, a custom R function was created to conduct iterative KW tests on each OTU to determine whether differences detected in class abundance among sites were driven by particular OTUs. Dunn’s post hoc tests with Bonferroni corrections were again used to determine pairwise differences among sites for significant KW tests. Specific OTUs are discussed if their relative abundance explained the results of the bacterial class comparisons.

Alpha diversity was calculated using the Shannon Index to compare the bacterial OTU diversity among sites within each community type (i.e., leaves, roots and rhizomes, and sediment). A KW test with a Bonferroni-corrected Dunn’s test was used for post hoc comparison to determine significant differences in bacterial diversity between sites.

## Results

### Summary of 16S rRNA Data

Amplicon DNA sequencing for seagrass leaves (243,198 total sequence reads), roots and rhizomes (191,817 total sequence reads), and sediment (94,328 total sequence reads) resulted in a total of 529,343 total sequence reads. These sequences were binned into 7759 distinct OTUs. The shortest sequence obtained was 150 bp and the longest was 670 bp with an average length of each sequence read being 435 ± 1.37 bp (Table 1).

**Table 1:** **16S** rRNA sequencing data of bacteria from plant sections and sediment from the sampling sites (from the Reference towards the Vent)

		Reference	Site 1	Site 2	Site 3	Vent
Leaves	Mean sequence reads (se)	22,136 (4874)	16,391 (4106)	14,072 (4587)	6475 (580)	18,535 (9721)
	Mean OTUs detected (se)	420 (57)	302 (19)	706 (105)	272 (51)	346 (61)
	Mean bp of OTU (se)	434.29 (2.25)	438.66 (2.5)	444.64 (1.26)	449.64 (1.91)	434.99 (2.1)
	Mean GC % (se)	53.89 (0.12)	54.21 (0.12)	53.6 (0.08)	53.53 (0.13)	53.74 (0.13)
Roots and rhizomes	Mean sequence reads (se)	12,596 (1137)	8,751 (871)	7,170 (921)	8,751 (871)	6,608 (2050)
	Mean OTUs detected (se)	592 (101)	765 (85)	711 (61)	565 (91)	642 (20)
	Mean bp of OTU (se)	445.66 (1.32)	452.14 (1.06)	452.42 (1.05)	455.13 (1.21)	447.34 (1.13)
	Mean GC % (se)	53.59 (0.08)	53.53 (0.07)	53.98 (0.07)	53.85 (0.08)	53.37 (0.07)
Sediment	Mean sequence reads (se)	5511 (542)	5891 (478)	4631 (793)	3669 (367)	5170 (686)
	Mean OTUs detected (se)	1214 (99)	1378 (123)	1038 (111)	988 (56)	809 (56)
	Mean bp of OTU (se)	457.82 (0.88)	458.01 (0.85)	457.76 (0.98)	459.7 (0.92)	458.43 (1.11)
	Mean GC % (se)	54.98 (0.06)	54.82 (0.06)	54.87 (0.06)	54.72 (0.06)	54.02 (0.08)

### Comparison Between Samples and Sites

**Fig. 2:**
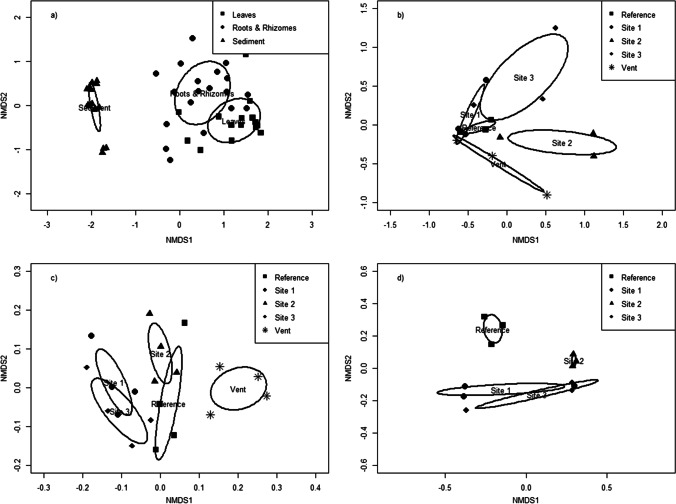
NMDS ordination plots of the bacterial community comparing (**a**) sample types of “Leaves” (black squares), “Roots and Rhizomes” (black circles), and “Sediment” (black triangles) (stress: 0.1321), (**b**) comparison of leaf bacterial communities among sites (stress: 0.1270), (**c**) comparison of root and rhizome bacterial communities among sites (stress: 0.1404), and (**d**) comparison of sediment bacterial communities among sites (site “Vent” was removed within the sediment plot as it was a major outlier, impeding the NMDS ordination plot from effectively communicating the comparison) (stress: 0.0574). Black circles represent a 95% confidence interval

There was a significant difference in bacterial communities detected among sample types (*F* = 14.061; *R*^2^ = 0.374; *p* < 0.001), with little to no overlap of sample types within the NMDS plot (Fig. 2a). A pairwise PERMANOVA showed that bacterial communities from each sample type were significantly different from the others (leaves vs. roots and rhizomes: *F* = 3.675, *R*^*2*^ = 0.100, *p* < 0.001; leaves vs sediment: *F* = 22.820, *R*^2^ = 0.449, *p* < 0.001; roots and rhizomes vs. sediment: *F* = 16.062, *R*^2^ = 0.327, *p* < 0.001).

When the bacterial communities were compared within sample types, there were no differences among sites for seagrass leaves (*F* = 1.395; *R*^2^ = 0.358; *p* = 0.092, Fig. 2b). Results of the PERMANOVA indicated that the bacterial community of the roots and rhizomes significantly differed among sites (*F* = 1.895; *R*^2^ = 0.336; *p* = 0.002) with some overlap within the NMDS ordination plot (Fig. 2c). Pairwise PERMANOVAs indicated that the bacterial community within roots and rhizomes were significantly different between the “reference” and “vent” sites (*F* = 2.348; *R*^2^ = 0.281; *p* = 0.048), “vent” and “site 2” (*F* = 2.209; *R*^2^ = 0.269; *p* = 0.042), “vent” and “site 3” (*F* = 2.825; *R*^2^ = 0.320; *p* = 0.019), and “site 2” and “site 3” (*F* = 1.551; *R*^2^ = 0.205; *p* = 0.032). When comparing the bacterial community of sediments among sites, bacterial OTUs found within the “vent” site were so specific to that location (*F* = 3.571; *R*^2^ = 0.588; *p* =  < 0.001) it prevented NMDS plot interpretation (Supplementary Fig. 1). Subsequent analyses removed the “vent” site (Fig. 2d) and still showed that the bacterial communities within the sediments significantly differed among the remaining sites (*F* = 1.683; *R*^2^ = 0.387; *p* = 0.004). The pairwise PERMANOVA of the bacterial community within the sediments, however, showed no significant differences among sites. Detailed pairwise PERMANOVA comparison results can be found in Supplemental Table 3.

### Bacterial OTU Diversity Indices

**Fig. 3:**
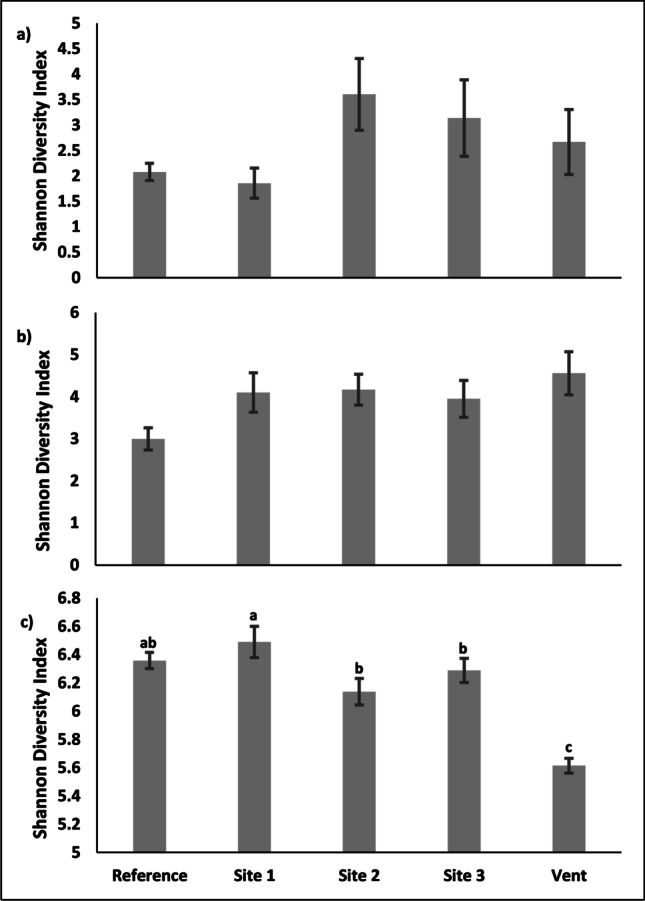
Comparison of average Shannon diversity index (± s.e.) among sites within (**a**) seagrass leaves, (**b**) seagrass roots and rhizomes, and (**c**) sediment samples. Letters identified above bars in (c) denote statistically similar and difference diversity indices among sites. Significance was determined by the post hoc Dunn’s test

Diversity, measured using the Shannon index, showed significant differences among sites for sediments only (*X*^2^ = 10.167, *df* = 4, *p* = 0.037; Fig. 3). No differences in alpha diversity were detected within the leaf or root bacterial communities (Fig. 3a,b). The post hoc analyses showed that the diversity of seagrass sediments was significantly lower in the “vent” site compared with that in “site 1” (*X*^*2*^ = 2.921; *p* = 0.017) (Fig. 3c).

### Bacterial Class Comparison

**Fig. 4:**
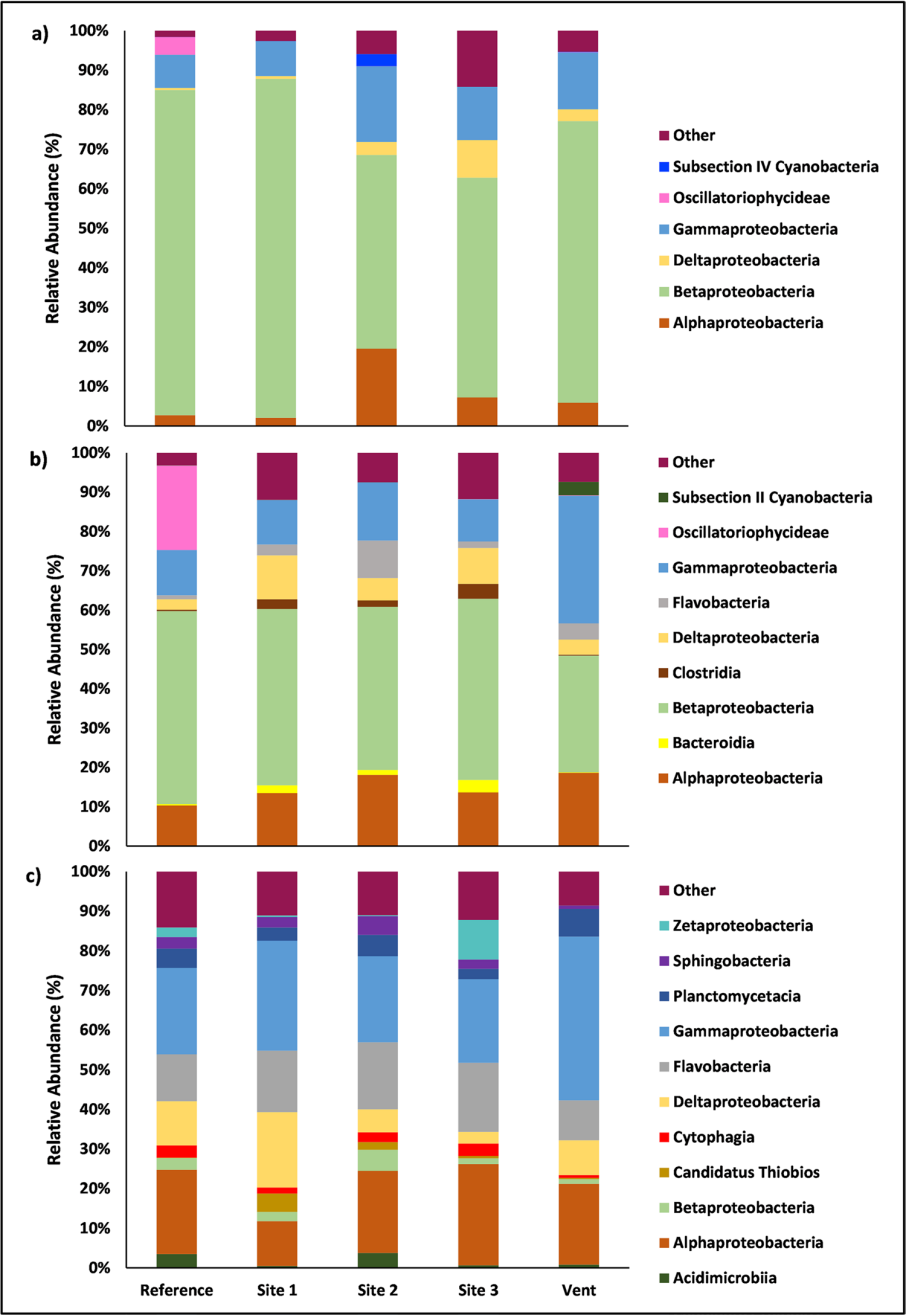
Relative abundance of bacterial classes found within samples for (**a**) Leaves, (**b**) Roots and Rhizomes, and (**c**) Sediments at each site. Class abundance under 3% were grouped into classification “other”

Six different bacterial classes dominated the leaf samples (Fig. 4a). *Betaproteobacteria* was the most abundant bacterial class at each site throughout the leaf community. The bacterial community of leaves at “site 1” had two dominant bacterial classes, *Betaproteobacteria* (85.77% SE ± 3.67) and *Gammaproteobacteria* (8.87% SE ± 0.98). *Alphaproteobacteria* and *Deltaproteobacteria* were major contributors to the bacterial communities of leaves at “site 2”, “site 3,” and “vent,” but not at the “reference” site nor at “site 1.” Out of the six dominant classes within the leaf community, two classes were dominant in only one site location. *Oscillatoriophycideae* was specific to the Reference site and an unknown *Cyanobacterium* was specific to “site 2.”

Only one of the dominant classes within the leaf community showed significant differences among sites that explained the pattern observed at the class level (Supplemental Fig. 2). *Oscillatoriophycideae* (*X*^2^ = 11.95, *df* = 4, *p* = 0.02) was significantly higher at the “reference” site compared to that at both “site 1” (*p* = 0.02) and “site 3” (*p* = 0.02). These significant differences were driven primarily by OTU 2 (o. *Oscillatoriales*) (*X*^2^ = 13.80, *df* = 4, *p* = 0.01), which showed significantly higher abundances at the “reference” site compared with all other sites (*p* = 0.02; Table 2). The average relative abundance of each significant bacterium within the leaf community can be found in Supplemental Fig. 2.

**Table 2:** Significant site differences between dominant bacterial classes for each sample type. Specific OTUs driving site differences are specified within classes (OTU numbers are specified within parenthesis). An arrow pointing up indicates the listed OTU had significantly higher relative abundance at the site compared with others. An arrow pointing down indicates the listed OTU had significantly lower relative abundance at the site compared with others. A negative sign indicates that either a specific OTU was not detected to explain differences among sites or the relative abundance of the OTU did not differ from other sites

Sample type	Class	OTU	Ref	Site 1	Site 2	Site 3	Vent
Leaf	*Oscillatoriophycideae*	*o. Oscillatoriales* (2)	↑	↓	↓	↓	↓
**Roots**	*Bacteroidia*	–	↓	–	–	↑	↓
	*Clostridia*	–	↓	–	–	↑	↓
	*Oscillatoriophycideae*	*o. Candidatus Thiobios* (2)	↑	↓	↓	↓	↓
	*unknown Cyanobacterium*	–	–	–	–	↓	↑
**Sediment**	*Acidimicrobiia*	*o. Acidimicrobiales* (152)	↑	–	–	–	↓
	*Candidatus Thiobios*	*o. Candidatus Thiobios* (46)	↓	–	–	–	↑
	*Gammaproteobacteria*	*g. Thiothrix (16)*	↓	↓	↓	↓	↑
		*sp. Balneatrix balneatrix (40)*	–	–	↓	–	↑
		*g. Balneatrix (1368)*	–	–	↓	–	↑
	*Sphingobacteria*	*–*	–	–	↑	–	↓
	*Zetaproteobacteria*	*–*	–	–	–	↑	↓

The bacterial community of the roots and rhizomes was dominated by nine different classes. Three of those classes were specific to the root samples: *Bacteroidia*, *Clostridia*, and *Subsection II Cyanobacteria*. Similar to seagrass leaves, *Betaproteobacteria* was heavily abundant in the roots and rhizomes (Fig. 4b). *Betaproteobacteria* was highest in abundance at all sites within the root community except at the “vent”, where *Gammaproteobacteria* was the most abundant (32.45% SE ± 13.09). Bacterial classes *Bacteroidia* (3.18% SE ± 0.59) and *Clostridia* (3.82% SE ± 1.34) were only dominant within “site 3” of the root community. *Subsection II Cyanobacteria* was only dominant within the root samples from the “vent” site. *Oscillatoriophycideae* was present within the “reference” site of the root community (21.49% SE ± 6.74).

The KW test revealed four bacterial classes significantly differed among sites within the root and rhizome community. These four groups were *Bacteroidia* (*X*^2^ = 13.67, *df* = 4, *p* = 0.01), *Clostridia* (*X*^2^ = 14.17, *df* = 4, *p* = 0.01), *Oscillatoriophycideae* (*X*^2^ = 12.86, *df* = 4, *p* = 0.01), and an *unknown Cyanobacterium* (*X*^2^ = 11.72, df = 4, *p* = 0.02). *Bacteroidia* showed a significantly higher abundance at “site 3” compared to the “reference” site (*p* = 0.02) and “vent” site (*p* = 0.01). *Clostridia* was significantly higher at “site 3” than at both the “reference” site (*p* = 0.02) and the “vent” site (*p* = 0.02). *Oscillatoriophycideae* was significantly higher at the “reference” site compared to “site 1” (*p* = 0.04), “site 2” (*p* = 0.01), and “site 3” (*p* = 0.04). Similar to leaves, OTU 2 (o. *Oscillatoriales*) showed significantly higher abundance at the “reference” site compared to all other sites (*p* < 0.01; Table 2), which explained the difference in *Oscillatoriophycideaea* detected at the class level (Supplemental Fig. 3). The *unknown Cyanobacterium* found at the “vent” site was significantly higher when compared with “site 3” (*p* = 0.01). *Bacteroidia*, *Clostridia*, and the *unknown Cyanobacterium* had no significant OTUs that differed among sites, which could have explained the differences detected at the class level. However, several other OTUs significantly differed among sites (detailed in Supplemental Fig. 3).

The bacterial community of the sediments was dominated by eleven different bacterial classes (Fig. 4c). Classes specific to the sediment community were *Acidimicrobiia*, *Candidatus Thiobios*, *Cytophagia*, *Planctomycetacia*, *Sphingobacteria*, and *Zetaproteobacteria*. *Gammaproteobacteria* was the most abundant at every sample location except “site 2” (20.73% SE ± 1.48), where *Alphaproteobacteria* was the most abundant (21.71% SE ± 1.55). Bacterial classes that were only detected at specific sites included *Acidimicrobiia* at the “reference” site (3.50% SE ± 0.37), *Cytophagia* at both “reference” (3.10% SE ± 0.13) and “site 2” (5.36% SE ± 3.90), *Candidatus Thiobios* at the “vent” site (6.92% SE ± 0.77), and both *Sphingobacteria* (3.75% SE ± 0.36) and *Zetaproteobacteria* at “site 2” (5.78% SE ± 0.64). *Planctomycetacia* was present in all sites except for the “vent” site and *Deltaproteobacteria* was present in all sites.

Seven of the eleven dominant classes showed significant differences among sites. *Acidimicrobiia* (*X*^2^ = 11.03, *df* = 4, *p* = 0.03) was significantly higher at the “reference” site when compared to that at the “vent” site (*p* = 0.01), primarily driven by a single OTU, *Acidimicrobiia*’s OTU 157 (o. *Acidimicrobiales*) (*p* = 0.02). Phylum *Proteobacteria’s*, *Candidatus Thiobios* (OTU 46) (*X*^2^ = 10.57, *df* = 4, *p* = 0.03) was significantly higher at the “vent” site compared with the “reference” site (*p* = 0.007). *Gammaproteobacteria* (*X*^2^ = 10.83, *df* = 4, *p* = 0.03) was significantly higher in abundance at the “vent” site when compared with that in the “reference” site (*p* = 0.04) and “site 2” (p = 0.01). *Gammaproteobacteria* had 3 OTUs within its classification that differed significantly among sites: OTU 16 (g*. Thiothrix*) (*X*^2^ = 13.80; *df* = 4; *p* = 0.01), OTU 40 (sp. *Balneatrix balneatrix*) (*X*^2^ = 10.22; *df* = 4; *p* = 0.04), and OTU 1368 (g. *Balneatrix*) (*X*^2^ = 11.07; *df* = 4; *p* = 0.03). OTU 16 was significantly higher at the “vent” site compared to that at all other locations (*p* = 0.02). OTU 40 was only significantly higher at the “vent” site compared to that at “site 2” (*p* = 0.02). OTU 1368 was also significantly higher at the “vent” site compared with that at “site 2” (*p* = 0.01; Table 2). The bacterial class *Planctomycetacia* (*X*^2^ = 10.83, *df* = 4, *p* = 0.03) was significantly different among sites, but the post hoc analysis failed to determine any significant differences among the sites. *Sphingobacteria* (*X*^2^ = 9.83, *df* = 4, *p* = 0.04) was significantly higher at “site 2” when compared with that at the “vent” site (*p* = 0.01). *Zetaproteobacteria* (*X*^2^ = 12.56, *df* = 4, *p* = 0.01) was significantly higher at “site 3” compared to that at the “vent” site (*p* = 0.01). *Planctomycetacia*, *Sphingobacteria*, and *Zetaproteobacteria* had no significant OTUs that differed among sites. The average relative abundance of each bacterial OTU within the sediment community that differed significantly among sites can be found in Supplemental Fig. 4.

## Discussion

In the present study, bacterial communities of *C. nodosa* leaves, roots rhizomes, and associated sediments were significantly different from each other. The bacterial community within the rhizosphere is influenced by plant metabolites [3] and may explain the slight overlap between the leaf and root samples in Fig. 1. However, environmental conditions specific to the site likely also influenced the below-ground microbiota of the seagrass roots and rhizomes. Indeed, there was a clear separation of these two communities, which were statistically different from each other, suggesting independent factors influence these two sample types. The bacterial community of the proximal sediment samples differed significantly from both of the seagrass sample types, had higher levels of diversity, and showed greater differences among sites. Interestingly, a similar pattern was apparent in a comparative study that took place in Bodega Bay, CA [35], suggesting these patterns may have a large geographical reach.

There were site-specific differences in bacterial communities of roots and rhizomes and sediments, but not in the leaf community, which remained consistent along the gradient. These results suggest that leaves may provide a more stable environment than roots, rhizomes, and sediment under changing levels of *p*CO_2_. However, our study did not incorporate water samples for microbiome analysis so this apparent stability may be a reflection of local bacterial communities within the aqueous environment. These results, however, do suggest that below-ground ecology might be influenced more by environmental change compared to above-ground ecology. Ettinger et al. (2017) showed a similar site-to-site difference in the sediment bacterial community, but not within the seagrass roots and rhizomes. Their study, however, characterized the microbial community in proximity to the seagrass patch and did not focus on changing environmental factors [35]. The addition of a strong environmental gradient and the lack of stability within the root microbiome found in our study compared to Ettinger et al. (2017) may suggest that under changing ocean conditions, the ability that seagrass leaves have to self regulate the root bacterial community might be restricted. It should be noted that our study, however, differed from Ettinger et al. (2017) in number of samples, sample processing (extraction kit and vortex period), primer selection (“universal” 515F and 806R), and sequencing depth. Mishra (2018) found an increase in the above to below-ground biomass ratio with increased *p*CO_2_ levels for *C. nodosa* at volcanic seeps in Greece and Italy, suggesting a greater impact of environmental factors on the below-ground ecology for this species of seagrass. The altered below-ground ecology in Mishra (2018) was also associated with a decline in *C. nodosa* survivability with increasing *p*CO_2_ due to reduced nutrient availability and soil toxicity [62]. However, differences in microbial communities among sites may not always be detrimental and may simply be a result of local adaptation to environmental conditions. Threats, like high *p*CO_2_, appear to have the greatest influence on the bacterial community of sediments, followed by the roots, and eventually the leaves if conditions become extreme [63, 64]. Seagrass leaves provide a niche for a specific bacterial community, dependant on chemical composition (sugars and secondary metabolites) [65], which did not change with *p*CO_2_. Duarte et al. (2018) showed marine macrophytes contain the ability to acclimate their hologenome under typically stressful ocean conditions [5]. Indeed, some marine macrophytes can regulate their bacterial community and its function under anthropogenic stressors as long as there is adequate nutrient availability [66, 67]. In the present study, the environmental pH gradient did not influence the diversity or composition of the bacterial community within the *C. nodosa* leaves as the bacterial communities associated with the *C. nodosa* leaves were most similar among sites. Currently, seagrass species that can regulate their microbiome under different environmental conditions appear to be limited to the leaves and, to a lesser extent, roots and rhizomes. Further investigation into the algal exudates potentially responsible for selecting bacteria should be considered to determine the degree of influence on the microbial community of the proximal sediment.

The present study showed that overall *Betaproteobacteria* (37.88%), *Gammaproteobacteria* (18.59%), and *Alphaproteobacteria* (14.05%) were the most abundant classes of bacteria among all samples at Levante Bay. Another study, which also quantified the bacterial communities of sediments within Levante Bay, showed the same dominant classes of bacteria to be the most abundant [68]. However, Kerfahi et al. (2014) also showed *Firmicutes, Bacteroidetes*, and *Actinobacteria* to be dominant as well. Alternatively, the present study detected several other taxa that were relatively common within the sediments that were not found in Kerfahi et al. (2014) including Zetaproteobacteria, Sphingobacteria, Planctomycetecia, Flavobacteria, Cytophagia, Candidatus Thiobios, and Acidimicrobia. Differences in the bacterial classes detected between these two studies could be ecologically or methodologically driven. Further longitudinal studies that utilize the same methods through time would better characterize the potential temporal changes of the sediment microbial structure within Levante Bay. Additionally, changes occur at different taxonomic scales. Analyzing bacteria at multiple taxonomic resolutions through time may elucidate further influences of environmental conditions on sediment microbiota.

The bacterial community of the leaves was heavily dominated by *Betaproteobacteria* regardless of the site, but this bacteria taxon was significantly lower within site 2 (pH 7.86) compared with the other sites. Similarly, a drop in *Betaproteobacteria* at site 2 was also recorded in the sediment. *Betaproteobacteria* plays an essential role in stabilizing the microbial community through the regulation of harmful algae [67]. *Betaproteobacteria* also is an ammonia-oxidizing bacteria (AOB) that plays an important role in nitrification. AOB abundance and nitrification rates have shown seasonal variability due to changing salinities [69]. However, the influence of pH on nitrification rates has not been studied for *C. nodosa*. The unique microbial community of the leaves and sediment sample types within site 2 suggests a concomitantly unique environmental condition within this location that warrants further exploration. Additionally, there was a decline in *Betaproteobacteria* at the vent site for all sample types, further indicating that this bacterium, a part of the *C. nodosa* core microbiome, may be susceptible to extreme *p*CO_2_ conditions.

Both the leaf and root microbial community showed significantly higher *Oscillatoriophycideae* — phylum Cyanobacteria — abundance within the “reference” site compared with the other sites with lower pH conditions. In addition, the biofilm on seagrasses within another naturally occurring CO_2_ vent in Papua New Guinea showed a significant reduction in the relative abundances and diversity of cyanobacteria closer to the vent compared with control sites [39]. Interestingly, cyanobacteria as part of a consortium of bacteria responsible for the coral disease black band, have significantly reduced abundance when under low pH conditions [70]. The results of the present study again suggest cyanobacterial abundances may be reduced under low pH conditions, although nutrient availability may also influence the presence of cyanobacteria [71], which was not accounted for in this study. Cyanobacteria are more common in oligotrophic conditions [72, 73], which could explain why we found a significantly higher abundance at the “reference” site compare to sites closer to the CO_2_ vents. Nitrogen-fixing cyanobacteria are prevalent on seagrass leaves. However, they are also found to inhabit other sections of the plant, which includes the rhizomes that do not receive light [13]. The ability of cyanobacteria to inhabit areas with low light could explain why there was a similar pattern between the leaves and roots and rhizomes for *Oscillatoriales* (Table 2). Further studies could identify whether the reduction in cyanobacteria under low pH conditions is a result of changing absolute abundances, increased abundance of other taxa, or more oligotrophic conditions further away from the CO_2_ vents.

The present study documented site-specific responses at both the bacterial class and OTU level, suggesting that small-scale environmental variations not captured within the study could be influencing the bacterial microbiome. There were several OTUs that significantly differed among sites (Supplemental Figs. 2, 3, 4) sites. However, only one OTU significantly contributed to differences at the bacterial class level within the leaf and root samples (*Oscillatoriales*). Differences among classes in sediment samples were driven by just five OTUs, o. *Acidimicrobiales*, o. *Candidatus Thiobios*, *g. Thiothrix, sp. Balneatrix balneatrix*, and *g. Balneatrix*. This suggests that a more detailed analysis at the OTU level could either reveal additional meaningful nuances within the data or lead to large levels of variation that mask trends at greater taxonomic levels. For instance, two OTUs belonging to o. *Candidatus Thiobios* and g. *Thiothrix* were significantly higher at the vent site. The higher abundance of these two OTUs is likely due to their sulfur oxidative properties, which are important for carbon fixation in *p*CO rich environments and are common bacteria surrounding hydrothermal vents [74, 75]. As a freshwater genus of bacteria, the higher abundance of g. *Balneatrix* at vent sites may be due to groundwater discharge from the Vulcano Porto Aquifer [76]. However, the association between the natural pH gradient and variability in each of these OTUs along a small geographical has not been well documented. The present study, as well as others [35], suggests that small geographic ranges can lead to significant changes within the bacterial communities of both seagrasses and their proximal sediments.

The species richness of the microbial community was not significantly different among sample types or sites, but previous studies showed that epibiont communities increase in species richness with declining pH levels creating a more complex microbial community [77]. Previous studies also showed that changes in pH can significantly affect the diversity of the microbial community within marine sediments [67, 78, 79]. In the present study, sediment samples were the most diverse compared to the other community types, although diversity significantly decreased within the low pH conditions of the vent site. The dominant bacteria *Gammaproteobacteria* nearly doubled in relative abundance under the extreme conditions at the vent site. The increase of *Gammaproteobacteria* could be due to the loss of rare and competing taxa. Similar to the present study, Kerfahi (2014) demonstrated a shift in the sediment bacterial community under low pH conditions at Levante Bay, Italy [68]. However, they found that the dominant bacteria *Gammaproteobacteria* remained stable in abundance, despite the shift in more rare taxa within the bacterial community with reduced pH. Site location must be considered, nevertheless, as there were differences in both site location and pH levels within the study sites. Despite these differences, our study and that of Kerfahi (2014) show stability within the sediment microbial community for dominant bacteria such as *Gammaproteobacteria* before extreme conditions are reached. Both studies also highlight the potential for disruption within the community of rare taxa [68]*.* Alternatively, Krause et al. (2012) suggest sediment microbial communities were less stable in the North Sea with moderate changes in pH resulting in significant alterations to the microbial assemblage [79]. Despite these differences, data suggest the sediment microbiome is less regulated than the leaves, roots, and rhizomes of seagrasses, perhaps because of the sediment’s separation from a biological host [2, 35].

Characterizing the different microbiome structures under natural environmental gradients, such as that in Levante Bay, is critical to providing insight into how a major component of organismal physiology, the host microbiome, will change under future ocean conditions. The results of the present study suggest *C. nodosa* may regulate its microbial community in the face of extreme pH conditions. The results also suggest that the surrounding environment may disrupt the microbiome of the seagrass root structures as well as the sediments themselves more so than the leaves. However, to date, there is no complementary study that assesses the bacterial turnover rates for *C. nodosa* with associated water samples through time. A study such as this could further elucidate the spatial and temporal variations of the microbiome within seagrass habitats*.* Characterizing the potential shifts in the microbial community of seagrasses under varying environmental conditions will be vital to determine the physiological ramifications of global change on this foundational taxonomic group.

## Supplementary Information

Below is the link to the electronic supplementary material.

Supplementary file1 (DOCX 2567 KB)

## Data Availability

Data on the 16S rRNA sequences analyzed in this study is available within the NCBI SRA database under BioProject ID: PRJNA623005.

## Code Availability

Code will not be made publicly available.
