# Supplementary material for: Instability and Stasis Among the Microbiome of Seagrass Leaves, Roots and Rhizomes, and Nearby Sediments Within a Natural pH Gradient
Source: Microb Ecol. 2021 Oct 1;84(3):703–16. doi: 10.1007/s00248-021-01867-9 (PMC9622545; doi:10.1007/s00248-021-01867-9)
Supplement: Supplementary file 1 — Supplementary file1 (DOCX 2567 KB) [file 248_2021_1867_MOESM1_ESM.docx]

**Supplementary Data**

**Instability and stasis among the microbiome of seagrass leaves, roots and rhizomes, and nearby sediments within a natural pH gradient**

**Microbial Ecology**

Raymond B. Banister^1^, Melbert Schwarz^1^, Maoz Fine^2,3^, Kim B. Ritchie^4^, Erinn M. Muller^1^

*^1^*Mote Marine Laboratory, Coral Health and Disease Program, Sarasota, FL USA

*^2^The Goodman Faculty of Life Sciences, Bar-Ilan University Ramat Gan 52900, Israel*

*^3^The Interuniversity Institute for Marine Science, P.O.B. 469, Eilat 88103, Israel*

*^4^Department of Natural Sciences University of South Carolina Beaufort, 801, Carteret St.
Beaufort, SC 29906*

**E-mail:** rbanister2019@my.fit.edu

Supplemental Figure 1. NMDS ordination plot of the bacterial OTU’s comparing sites within the ‘Sediment’ *community* (stress: 0.068) (site 'Vent' was removed for Figure 2 in the manuscript as it was a major outlier, impeding the NMDS ordination plot from effectively communicating the comparison). Open circles represent a 95 percent confidence, but are not visible due to the ‘Vent’ site outlier

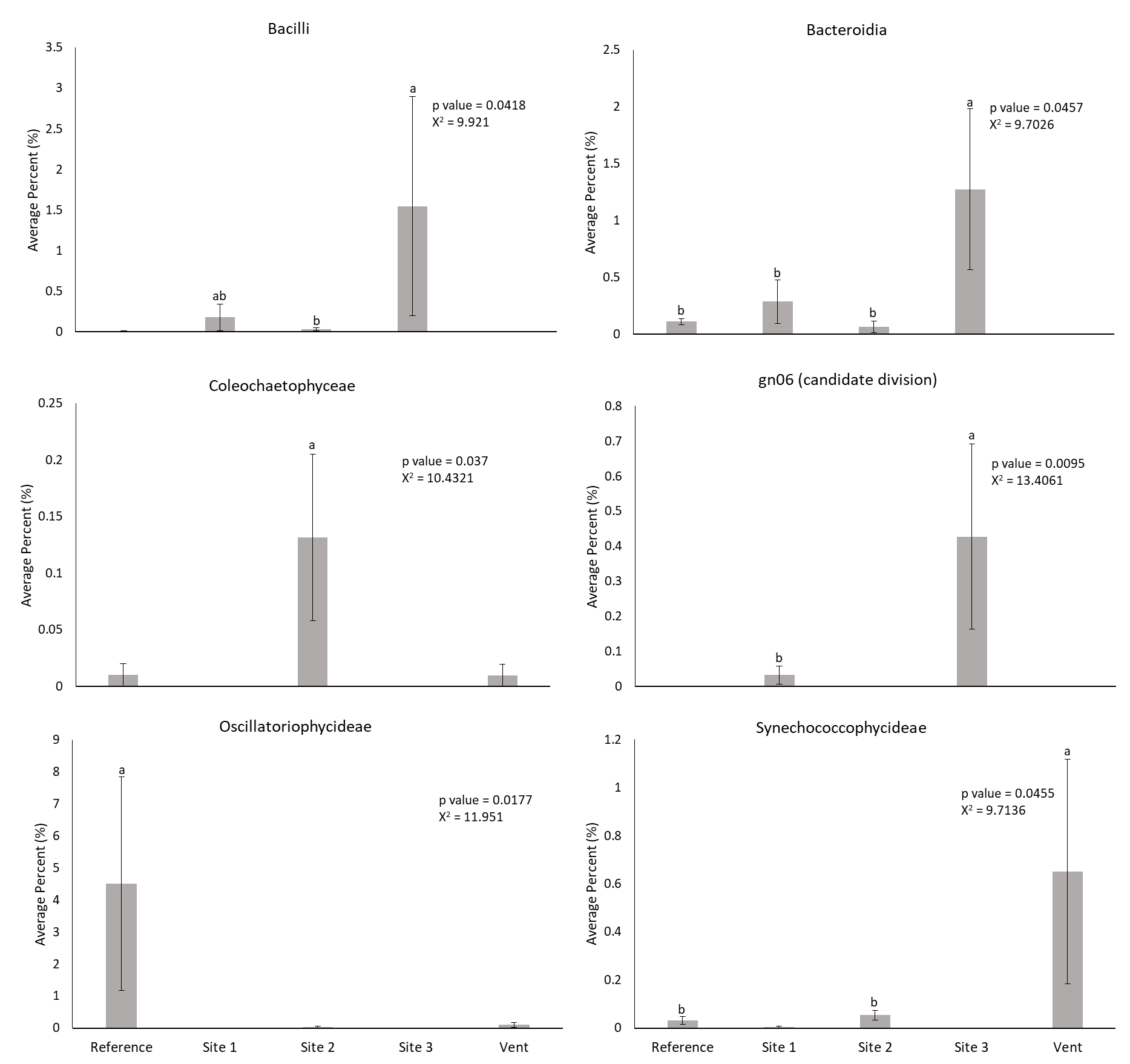


Supplemental Figure 2. A bar graph comparison showing the average relative abundance (+s.d.) of each bacteria that significantly differed among sites within the seagrass leaf samples (determined using K.W. Test). Letters above the bars indicate those that significantly differed from each other.

Supplemental Figure 3. A bar graph comparison showing the average relative abundance (+s.d.) of each bacteria that significantly differed among sites within the seagrass root/rhizome samples (determined using K.W. Test). Letters above the bars indicate those that significantly differed from each other.


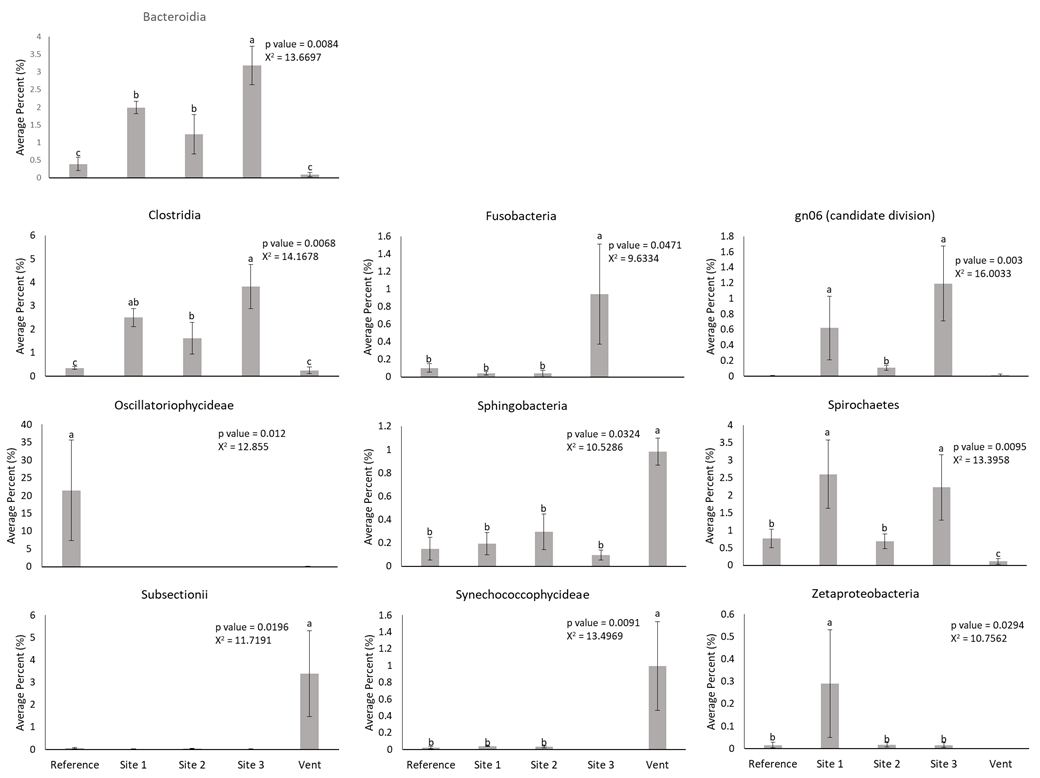


Supplemental Figure 4. A bar graph comparison showing the average relative abundance (+s.d.) of each bacteria that significantly differed among sites within the proximal sediment samples (determined using K.W. Test). Letters above the bars indicate those that significantly differed from each other.


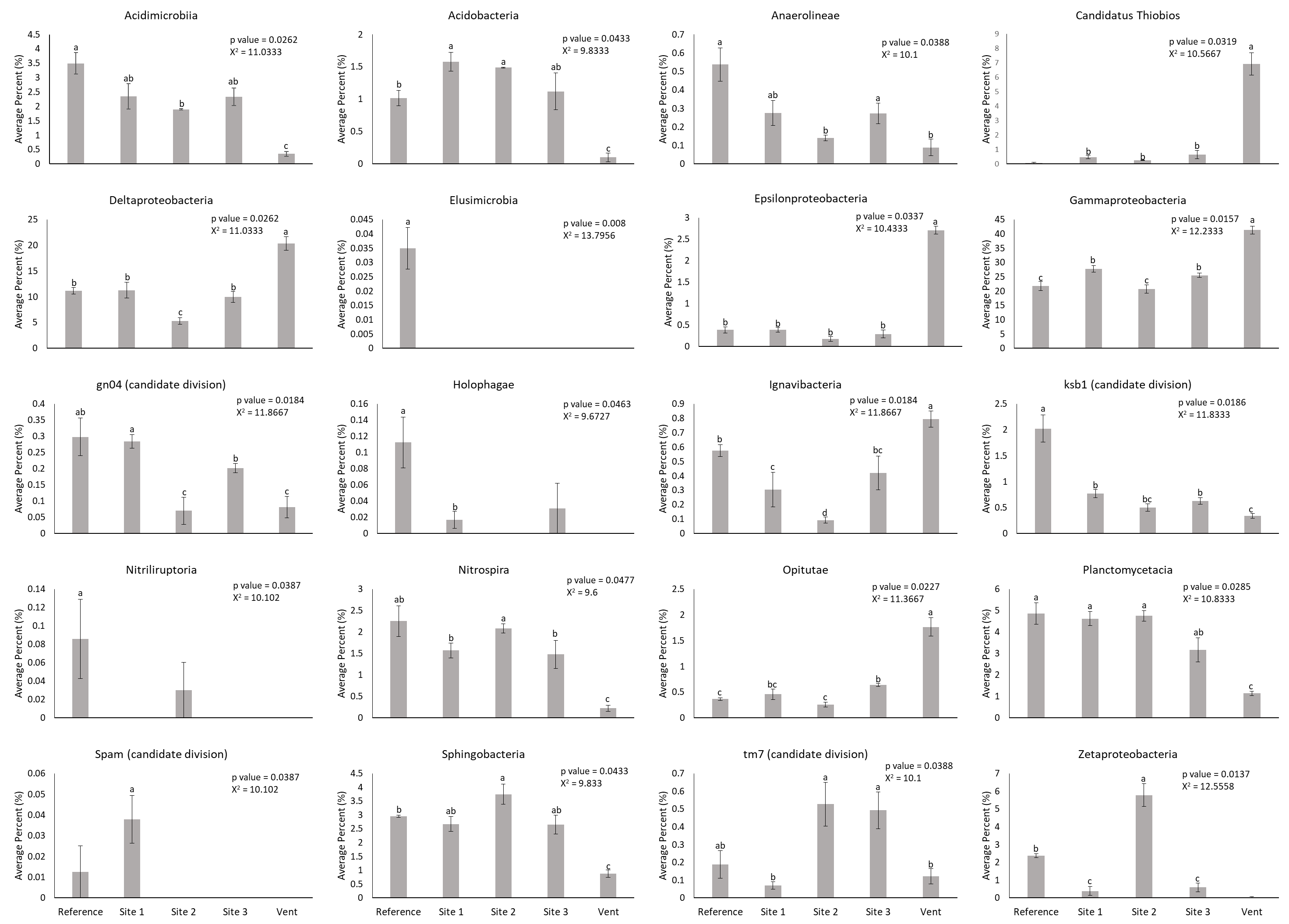


Supplemental Table 1 Physiochemical data from Milazzo et al. (2016) [54] and Vizzini et al. (2019) [55] for control and low pH sites that correspond based on proximity with Site 3 (pH 7.44) and Site 1 (pH 8.03) in the current study.

| Physiochemical Property | Average ± Standard Error | Corresponding Site | Reference Study |
| --- | --- | --- | --- |
| Total Alkalinity (mmol kg^−1^) | 2.5 | Site 3 (pH 7.44) | [54] |
|  | 2.5 | Site 1 (pH 8.03) | [54] |
| *p*CO2 (μatm; mean + SD) | 1180 ± 153 | Site 3 (pH 7.44) | [54] |
|  | 421 ± 15 | Site 1 (pH 8.03) | [54] |
| Filamentous Algae (‰; mean + SD) | N/A | Site 3 (pH 7.44) | [55] |
|  | −23.44 ± 0.17 | Site 1 (pH 8.03) | [55] |
| Suspended Particulate Organic Matter (‰; mean + SD) | −24.76 ± 0.14 | Site 3 (pH 7.44) | [55] |
|  | −20.87 ± 1.05 | Site 1 (pH 8.03) | [55] |
| Macroalgae (‰; mean + SD) | −19.75 ± 0.77 | Site 3 (pH 7.44) | [55] |
|  | N/A | Site 1 (pH 8.03) | [55] |
| Microbial Mats (‰; mean + SD) | −26.11 ± 0.91 | Site 3 (pH 7.44) | [55] |
|  | N/A | Site 1 (pH 8.03) | [55] |
| Dry Bulk Density of Sediment (g cm^−3^; mean + SD) | 1.54 ± 0.06 | Site 3 (pH 7.44) | [55] |
|  | 1.55 ± 0.02 | Site 1 (pH 8.03) | [55] |
| Porosity (φ; mean + SD) | 0.38 ± 0.01 | Site 3 (pH 7.44) | [55] |
|  | 0.42 ± 0.03 | Site 1 (pH 8.03) | [55] |
| Silt and Clay (%; mean + SD) | 1.4 ± 0.5 | Site 3 (pH 7.44) | [55] |
|  | 2.8 1.0 ± 0.1 | Site 1 (pH 8.03) | [55] |
| δ13C (‰; mean + SD) | −22.69 ± 1.94 | Site 3 (pH 7.44) | [55] |
|  | −21.94 ± 0.21 | Site 1 (pH 8.03) | [55] |
| C/N (mean + SD) | 3.66 ± 2.48 | Site 3 (pH 7.44) | [55] |
|  | 10.54 ± 1.72 | Site 1 (pH 8.03) | [55] |

Supplemental Table 2. Classification scheme used to identify operational taxonomic units to the lowest taxon

| **Identity to reference sequence** | **Identity Designation** |
| --- | --- |
| > 97% | Species |
| Between 97% and 95% | (unclassified Genus) |
| Between 95% and 90% | (unclassified Family) |
| Between 90% and 85% | (unclassified order) |
| Between 85% and 80% | (unclassified class) |
| Between 80% and 77% | (unclassified phylum) |
| < 77% | (unknown) |

Supplemental Table 3. Pairwise PERMANOVA comparing the bacterial community among sites within leaves, roots and rhizomes, and sediment with vent removed

| Site | Site Comparison for All Types | | | | | |
| --- | --- | --- | --- | --- | --- | --- |
| Comparison | "Signif. codes: 0‘***’ 0.001 ‘**’ 0.01 ‘*’ 0.05 ‘.’ 0.1 ‘ ’ 1" | | | | | |
| for |  | pairs | F.Model | R2 | p.value | p.adjusted sig |
| Types | 1 | Reference vs Vent | 2.3899549 | 0.11721237 | 0.045 | 0.45 |
|  | 2 | Reference vs Site 1 | 0.8663058 | 0.04591814 | 0.412 | 1 |
|  | 3 | Reference vs Site 2 | 1.1462484 | 0.05986804 | 0.26 | 1 |
|  | 4 | Reference vs Site 3 | 1.0553961 | 0.05538568 | 0.286 | 1 |
|  | 5 | Vent vs Site 1 | 2.1553583 | 0.10693724 | 0.049 | 0.49 |
|  | 6 | Vent vs Site 2 | 2.0887521 | 0.1039762 | 0.046 | 0.46 |
|  | 7 | Vent vs Site 3 | 1.9855982 | 0.09935145 | 0.071 | 0.71 |
|  | 8 | Site 1 vs Site 2 | 0.9676476 | 0.05101569 | 0.34 | 1 |
|  | 9 | Site 1 vs Site 3 | 0.6417477 | 0.0344253 | 0.714 | 1 |
|  | 10 | Site 2 vs Site 3 | 0.809045 | 0.04301361 | 0.503 | 1 |
| Leaves | 1 | Reference vs Site 1 | 1.8893432 | 0.3208071 | 0.2 | 1 |
|  | 2 | Reference vs Site 2 | 2.063384 | 0.3403024 | 0.2 | 1 |
|  | 3 | Reference vs Site 3 | 1.4726402 | 0.2690914 | 0.1 | 1 |
|  | 4 | Reference vs Vent | 1.4006974 | 0.2593549 | 0.2 | 1 |
|  | 5 | Site 1 vs Site 2 | 2.1606529 | 0.3507182 | 0.1 | 1 |
|  | 6 | Site 1 vs Site 3 | 1.3039345 | 0.2458429 | 0.2 | 1 |
|  | 7 | Site 1 vs Vent | 1.6063238 | 0.28652 | 0.2 | 1 |
|  | 8 | Site 2 vs Site 3 | 0.8807966 | 0.1804616 | 0.6 | 1 |
|  | 9 | Site 2 vs Vent | 1.3089401 | 0.2465539 | 0.2 | 1 |
|  | 10 | Site 3 vs Vent | 1.0943713 | 0.2148197 | 0.4 | 1 |
| Roots and Rhizomes | 1 | Reference vs Vent | 2.347858 | 0.2812528 | 0.048 | 0.48 |
|  | 2 | Reference vs Site 1 | 1.586258 | 0.2090962 | 0.177 | 1 |
|  | 3 | Reference vs Site 2 | 1.339325 | 0.1824862 | 0.266 | 1 |
|  | 4 | Reference vs Site 3 | 1.665039 | 0.2172251 | 0.151 | 1 |
|  | 5 | Vent vs Site 1 | 2.459123 | 0.2907066 | 0.07 | 0.7 |
|  | 6 | Vent vs Site 2 | 2.209368 | 0.2691276 | 0.042 | 0.42 |
|  | 7 | Vent vs Site 3 | 2.825177 | 0.320127 | 0.019 | 0.19 |
|  | 8 | Site 1 vs Site 2 | 1.499292 | 0.1999245 | 0.081 | 0.81 |
|  | 9 | Site 1 vs Site 3 | 1.164624 | 0.162552 | 0.206 | 1 |
|  | 10 | Site 2 vs Site 3 | 1.550664 | 0.2053679 | 0.032 | 0.32 |
| Sediment | 1 | Reference vs Vent | 9.804823 | 0.7102462 | 0.1 | 1 |
|  | 2 | Reference vs Site 1 | 1.740514 | 0.3031983 | 0.1 | 1 |
|  | 3 | Reference vs Site 2 | 2.853429 | 0.4163506 | 0.1 | 1 |
|  | 4 | Reference vs Site 3 | 1.859596 | 0.3173591 | 0.1 | 1 |
|  | 5 | Vent vs Site 1 | 6.285036 | 0.6110854 | 0.1 | 1 |
|  | 6 | Vent vs Site 2 | 9.34829 | 0.7003361 | 0.1 | 1 |
|  | 7 | Vent vs Site 3 | 5.803967 | 0.5920019 | 0.1 | 1 |
|  | 8 | Site 1 vs Site 2 | 1.814203 | 0.3120296 | 0.1 | 1 |
|  | 9 | Site 1 vs Site 3 | 0.820806 | 0.1702632 | 0.7 | 1 |
|  | 10 | Site 2 vs Site 3 | 1.426772 | 0.2629136 | 0.1 | 1 |
|  | 1 | Reference vs Site 1 | 1.740514 | 0.3031983 | 0.1 | 0.6 |
|  | 2 | Reference vs Site 2 | 2.853429 | 0.4163506 | 0.1 | 0.6 |
| Sediment | 3 | Reference vs Site 3 | 1.859596 | 0.3173591 | 0.1 | 0.6 |
| no Vent | 4 | Site 1 vs Site 2 | 1.814203 | 0.3120296 | 0.1 | 0.6 |
|  | 5 | Site 1 vs Site 3 | 0.820806 | 0.1702632 | 0.7 | 1 |
|  | 6 | Site 2 vs Site 3 | 1.426772 | 0.2629136 | 0.1 | 0.6 |
